# Neuropsychosocial–Spiritual Correlates of Serene Abstinence in Adults with Alcohol Use Disorder Receiving Community-Based Recovery Care in Romania: A Longitudinal Observational Study Protocol (SER-ABS-AUD)

**DOI:** 10.3390/jcm15218260

**Published:** 2026-10-23

**Authors:** Alexandra-Maria Găină, Alexandra Hasnaș, Marcel-Alexandru Găină, Ionela-Ioana Darcaci, Bogdan-Victor Ștefănescu, Cristina-Gabriela Șchiopu, Bogdan-Marian Tarcău, Liviu-Adrian Măgurianu, Andreea-Silvana Szalontay

**Affiliations:** 1 Doctoral School, Grigore T. Popa University of Medicine and Pharmacy, 16 Universitatii Street, 700115 Iasi, Romania; alexandra.gaina@email.umfiasi.ro (A.-M.G.); bogdan-victor.c.stefanescu@d.umfiasi.ro (B.-V.Ș.); cristina-gabriela.schiopu@umfiasi.ro (C.-G.Ș.); bogdan-marian.tarcau@d.umfiasi.ro (B.-M.T.); 2 Association of Recovering Alcoholics (ACAR) Don Orione Residential Centre, Rediu 21/C, 700546 Iași, Romania; acar.donorione@gmail.com (A.H.); acar@alcoologia.ro (I.-I.D.); 3 Third Medical Department, Grigore T. Popa University of Medicine and Pharmacy, 700115 Iași, Romania; andreea.szalontay@umfiasi.ro; 4 “Gheorghe Zane” Institute for Economic and Social Research, Romanian Academy, 700481 Iași, Romania; liviu.magurianu@acadiasi.ro; 5 Socola Institute of Psychiatry, Bucium 36, 700282 Iași, Romania

**Keywords:** alcohol use disorder, sustained abstinence, recovery, MCMI-IV, quality of life, spirituality, social support, community-based care, Romania

## Abstract

**Background/Objectives:** Alcohol use disorder (AUD) contributes substantially to premature mortality and disability in Romania. Recovery is commonly evaluated according to abstinence status, with less attention to psychological functioning, social support, spiritual well-being, and quality of life. SER-ABS-AUD (ClinicalTrials.gov identifier NCT07561749) examines ‘serene abstinence’ as a preliminary multidimensional recovery construct among adults with AUD receiving community-based recovery support. **Methods:** This single-center, non-interventional, mixed retrospective–prospective longitudinal cohort study aims to enroll 70 adults aged 18–70 years with documented AUD, at least 12 consecutive months of continuous alcohol abstinence before enrollment, and an archived pre-enrollment MCMI-IV profile. Following written informed consent at the current T2 study visit, archived T1 MCMI-IV data and available clinical information will be extracted, and the MCMI-IV and supplementary recovery-outcome battery will be administered prospectively at T2. The principal longitudinal outcome is within-person change in prespecified MCMI-IV scales between archived T1 and current T2 assessment. The secondary exploratory Serene Abstinence Index combines MCMI-IV persistent depression change with current T2 natural reward response, spiritual well-being, and quality of life. Supplementary T2 measures will be interpreted as current outcomes or concurrent exploratory correlates, not as repeated longitudinal measures. **Results:** Recruitment is ongoing; no participant-level outcome data are reported in this protocol. **Conclusions**: SER-ABS-AUD will estimate longitudinal MCMI-IV change and characterize multidimensional recovery in a Romanian community-based AUD setting. Findings will be hypothesis-generating and will require replication and external validation in larger, independent, multicentre cohorts.

## 1. Introduction

Alcohol use disorder (AUD) remains one of the leading preventable causes of morbidity and premature mortality in Europe. Globally, an estimated 400 million people live with AUD, of whom 209 million meet criteria for alcohol dependence; alcohol consumption accounted for 2.6 million deaths (4.7% of all deaths) in 2019 [1]. Romania ranks among the highest-burdened countries in the European Union. Alcohol use was responsible for approximately 5% of all deaths nationally in 2021, exceeding the OECD average of 3% [2]. Romania continues to face important gaps in the continuity of care for alcohol-related disorders, particularly at the transition from acute treatment to longer-term recovery support [2]. Beyond its psychiatric and social burden, chronic AUD may be associated with neurological sequelae, including withdrawal seizures, alcoholic polyneuropathy, cerebellar dysfunction, and, in severe cases, Wernicke–Korsakoff spectrum disorders; these conditions can impair physical functioning and the psychological stability needed for sustained recovery [3,4,5].

Even when abstinence is achieved and sustained, binary classification as abstinent versus relapsed may not capture important differences in psychological functioning and lived recovery. In this protocol, the terms ‘struggle abstinence’ and ‘serene abstinence’ are used as investigator-defined working labels for contrasting recovery experiences rather than as diagnostic categories or validated clinical endpoints. ‘Struggle abstinence’ refers to sustained sobriety accompanied by persistent craving, residual anhedonia, hypervigilance, or marked subjective effort. ‘Serene abstinence’ refers to sustained sobriety accompanied by lower anhedonia-related symptom burden, greater responsiveness to ordinary non-alcohol rewards, renewed meaning, and better current quality of life. These descriptions are intended to motivate exploratory operationalization; they do not imply a validated dichotomy, and recovery may occur along a continuum. Anhedonia in this context has a well-characterized psychobiological substrate. Withdrawal and early abstinence are associated with hypoactivity of the meso-cortico-limbic dopaminergic reward system. Early abstinence and substance-use disorders have been associated with altered reward processing, reduced responsiveness to natural reinforcers, and persistent anhedonia. These reward-related disturbances may contribute to craving and relapse vulnerability, although the present study does not measure dopaminergic function directly [6,7]. This distinction has direct clinical relevance, since individuals experiencing persistent distress during abstinence may have greater unmet clinical and psychosocial support needs; this proposition will not be tested as a relapse-risk outcome in the present protocol, yet the distinction has not been operationalized or studied longitudinally in a Romanian cohort.

This limitation is not merely conceptual: improvements in quality of life following addiction treatment have been associated with reductions in substance use and may capture broader functional change not reflected by abstinence status alone. This evidence supports the inclusion of subjective quality of life, assessed in the present study using the Quality of Life Inventory (QOLI), as one component of the Serene Abstinence Index [8]. In this protocol, QOLI is interpreted as a prospectively assessed T2 recovery indicator rather than as a standardized repeated T1–T2 measure of quality-of-life variation.

Depressive symptoms occurring in the context of chronic alcohol use may differ clinically and biologically from primary major depressive disorder. This distinction supports the present protocol’s separate assessment of dysthymic symptoms, anhedonia-related reward disturbance, and broader affective symptom burden rather than treating all depressive manifestations as a single undifferentiated construct [9].

The biopsychosocial–spiritual model provides a framework for examining recovery as a multidimensional process involving biological functioning, psychological symptoms, social reintegration, and spiritual well-being. Longitudinal research in community-linked addiction care supports the relevance of assessing outcomes beyond substance use alone, including mental health, subjective well-being, social functioning, and employment-related outcomes [10].

Evidence regarding spirituality in recovery suggests that meaning, purpose, connectedness, and spiritual coping may be relevant to recovery experiences and substance-use outcomes; however, their effects are likely to vary across individuals and treatment settings [11,12,13]. Similarly, social support and participation in recovery-oriented communities may support treatment engagement and sustained recovery [14,15].

The Millon Clinical Multiaxial Inventory-IV (MCMI-IV) is used in this protocol to characterize selected personality patterns and clinical-syndrome dimensions relevant to recovery [16]. The prespecified scales of interest are alcohol use (Scale B), persistent depression (Scale D), generalized anxiety (Scale A), avoidant personality pattern (Scale 2A), dependent personality pattern (Scale 3), and masochistic personality pattern (Scale 8B). Scale D is used as an indicator of persistent depressive symptom burden, whereas Scale A is used to characterize generalized anxiety symptoms. The selected personality-pattern scales are examined as candidate correlates of social withdrawal, dependency-related interpersonal difficulties, and self-defeating interpersonal patterns. MCMI-IV scores will not be interpreted as direct indicators of specific neurobiological mechanisms. The relevance of personality dimensions to AUD is also supported by network-analytic evidence linking externalizing personality traits with AUD diagnostic criteria [17]. More broadly, the frequent overlap between substance-use disorders and mood, anxiety, trauma-related, and personality disorders supports the decision to model comorbid psychiatric conditions explicitly as covariates rather than to treat depressive or anxiety symptoms as isolated recovery markers [18]. The present protocol therefore examines MCMI-IV personality and clinical-syndrome dimensions as exploratory correlates of recovery trajectories; these measures are not used to establish causal mechanisms or formal psychiatric diagnoses.

Romania has a well-established, internationally accredited community recovery network built on the Hudolin Club methodology, first introduced in Iași in 2004 through the Association of Alcoholics in Recovery Clubs “Don Orione” (ACAR). ACAR operates a residential social rehabilitation center accredited by the Romanian Ministry of Labour and Social Justice since 2006, is affiliated with the World Association of Clubs of Alcoholics in Treatment (WACAT), and integrates the affected person and their family within peer-led support Clubs that address the physical, psychological, social, and spiritual dimensions of recovery in an ecological, community-oriented model rather than a purely psychiatric one. This model, sustained in Romania since 2004 and expanded to a second residential center and multiple regional support Clubs, provides a naturalistic setting in which to examine within-person MCMI-IV change between archived routine-care assessment and current enrollment assessment, together with multidimensional current recovery characteristics.

The objective of this protocol is to describe SER-ABS-AUD, a mixed retrospective–prospective longitudinal observational study that will: (1) characterize the sociodemographic, clinical, and MCMI-IV profile of a Romanian community-recovery AUD cohort; (2) estimate within-person change in prespecified MCMI-IV scales between archived T1 and current T2 assessment; (3) estimate the proportion meeting the strict and alternative exploratory Serene Abstinence Index definitions at T2; (4) examine transparent univariable associations between eligible archived T1 variables and strict SAI status, followed, only if supported by the observed event count, by a restricted penalized model; and (5) describe current T2 biological, psychological, social, spiritual, cognitive, and quality-of-life indicators and their concurrent associations with outcomes that do not mathematically include the same variables.

The principal exploratory hypothesis is that higher archived T1 MCMI-IV generalized anxiety (A) and avoidant personality-pattern (2A) scores will be associated with lower odds of meeting the strict SAI definition at T2. Duration of continuous abstinence before enrollment is retained as the third prespecified variable in the restricted adjusted model. Because MCMI-IV persistent depression change is part of the SAI definition, archived T1 persistent depression (D) will not be entered as an independent predictor of the composite. Current T2 spiritual well-being, social support, natural reward response, and quality of life will be analyzed as concurrent correlates only in analyses whose outcome does not contain the same construct.

## 2. Materials and Methods

### 2.1. Study Design and Registration

SER-ABS-AUD is a single-center, non-interventional, mixed retrospective–prospective longitudinal cohort study. The protocol was developed with reference to the STROBE Statement for cohort studies and the RECORD extension for observational studies using routinely collected health data; RECORD applies specifically to the archived routine-care component [19,20].

Study enrollment and the current T2 research assessment occur at the same study visit, after participants have completed at least 12 consecutive months of continuous alcohol abstinence. Following written informed consent, routinely collected pre-enrollment MCMI-IV profiles and available clinical information are extracted retrospectively as archived T1 data. The MCMI-IV and the supplementary biopsychosocial–spiritual recovery battery are then administered prospectively at the current T2 study visit. Thus, T1 and T2 identify assessment occasions rather than two post-enrollment research visits. No supplementary standardized research battery was administered at archived T1, and no additional 12-month prospective post-enrollment assessment is part of the present protocol. The elapsed time between archived T1 MCMI-IV assessment and current T2 assessment will be recorded for every participant and reported descriptively.

The study was approved by the Institutional Review Board of Grigore T. Popa University of Medicine and Pharmacy, Iași, Romania (approval no. 733, 9 March 2026), following a favorable opinion from the Ethics Committee of the Institute of Psychiatry Socola, Iași (no. 34565, 17 December 2025). The study is registered on ClinicalTrials.gov (NCT07561749; enrollment by invitation) [21]. Recruitment began on 20 April 2026. The registry record was updated to match the clarified assessment timing, outcome hierarchy, eligibility criteria, and anticipated completion dates. Any substantive protocol amendment will be submitted to the relevant ethics bodies in accordance with institutional requirements.

### 2.2. Setting

The study is conducted at the Association of Alcoholics in Recovery Clubs “Don Orione” (ACAR), Iași, Romania, and its affiliated residential recovery setting. ACAR provides community-based support for individuals with alcohol-related problems and their families through Club-based meetings and recovery-oriented activities addressing biological, psychological, social, and spiritual dimensions of functioning. SER-ABS-AUD is a single-center study recruiting through the active Iași recovery setting and affiliated support groups; participants may also be identified through referral pathways from the Institute of Psychiatry “Socola”, Iași, Romania, following inpatient detoxification. Although the Hudolin Club methodology is historically and organizationally distinct from formal therapeutic community treatment, longitudinal evidence from residential and community-linked addiction settings supports the relevance of evaluating biopsychosocial outcomes alongside substance-use outcomes over time [10].

#### Multidisciplinary Care and Research Team

**Figure 1:**
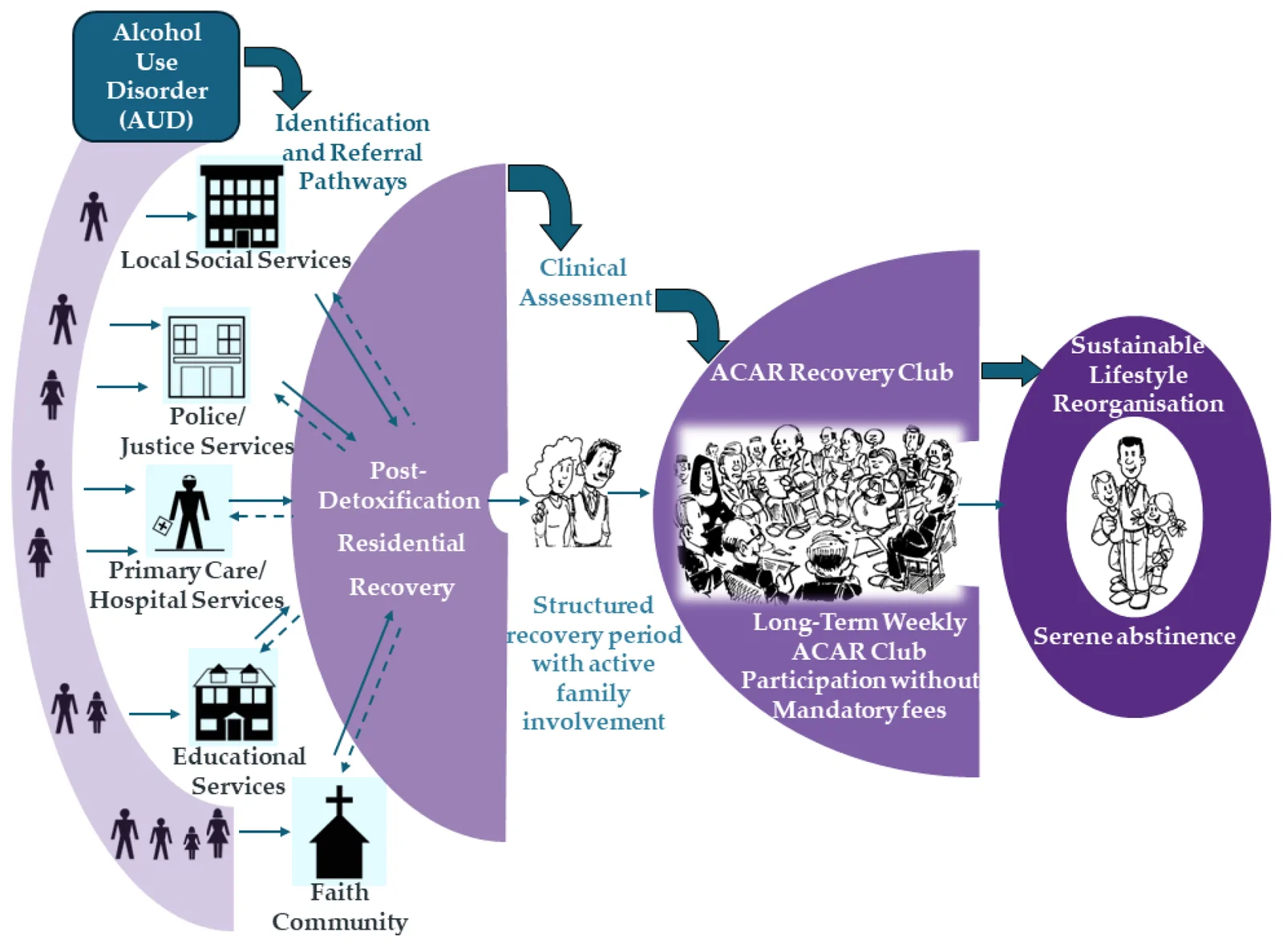
Community-based recovery pathway in the ACAR–Don Orione model.

Usual clinical and community recovery care at ACAR Don Orione is delivered by a stable multidisciplinary team whose composition reflects the biopsychosocial–spiritual model underlying the present protocol. Research procedures are conducted separately in accordance with the study protocol. Figure 1 illustrates the community-based recovery pathway within the ACAR Don Orione model, including identification and referral, clinical assessment and referral coordination, post-detoxification residential recovery with family involvement, and longer-term ACAR Club participation. These clinical and community recovery services constitute usual care and are not delivered, allocated, or modified as part of the SER-ABS-AUD research protocol. Alexandra-Maria Găină (A.-M.G.), first author of this protocol and a volunteer neurologist within the ACAR network, contributes to the identification and clinical evaluation of alcohol-related neurological complications, including withdrawal seizures, alcoholic polyneuropathy, cognitive impairment, and, where clinically indicated, Wernicke–Korsakoff spectrum presentations. These conditions may interfere with physical recovery and the quality of abstinence. To minimize perceived pressure and role-related measurement bias, written informed consent is obtained by trained study personnel who are not responsible for the participant’s routine clinical care. Research assessments are assigned to a non-treating assessor wherever staffing permits. If clinician–assessor overlap cannot be avoided, the overlap is recorded prospectively in the delegation and role-overlap logs and is addressed through standardized administration, independent verification of MCMI-IV item entry, validity indicators, and BR scores, and a prespecified sensitivity analysis. The frequency of any previous or current clinical relationship will be documented and reported. The principal anticipated overlap concerns a subset of female participants who may previously have received routine psychological support from A.H. Each participant’s assessor, the nature of any prior clinical relationship, and the mitigating procedures used are recorded before database lock. Personnel conducting independent score verification will not be provided with archived T1 scores, supplementary T2 outcome scores, or derived SAI status.

Marcel-Alexandru Găină (M.-A.G.), corresponding author of this protocol and volunteer psychiatrist, has provided voluntary psychiatric assistance to ACAR beneficiaries for the past eight years, with a clinical role focused on the recognition and management of psychiatric comorbidities, emotional distress, residual craving-related suffering, and relapse vulnerability in individuals engaged in long-term community recovery. Together, these neurological and psychiatric perspectives provide clinical context for interpreting medical and psychiatric comorbidity in recovery. In the research protocol, MCMI-IV persistent depression (Scale D) and generalized anxiety (Scale A) are prespecified clinical-syndrome variables, assessed retrospectively at T1 and repeated prospectively at T2. Alexandra Hasnaş (A.H.), clinical psychologist and coordinator of the residential center’s women’s unit, contributes to T2 administration and scoring of the MCMI-IV and supplementary recovery-outcome battery for female participants. The same prespecified administration, scoring, and data-entry procedures are applied across participants; the assessor responsible for male participants is specified in the study delegation log. Ionela-Ioana Darcaci (I.-I.D.), clinical psychologist and psychotherapist, coordinates the Iași residential center and oversees the psychological and psychotherapeutic components of the recovery program for all participants. Iulian Butnaru (I.B.), President of ACAR “Don Orione” and a Catholic priest, was responsible until August 2026 for the spiritual dimension of the program, including pastoral accompaniment, spiritual community activities, and supervision of the Club network’s spiritual counseling.

This multidisciplinary setting provides relevant clinical, psychological, social, and spiritual context for interpretation of recovery experiences. The exploratory Serene Abstinence Index integrates longitudinal MCMI-IV persistent depression (Scale D) change with prospectively assessed T2 recovery indicators; it is an investigator-defined research construct and does not represent a clinical diagnosis or a service-delivery assessment.

### 2.3. Participants

Eligible participants are adults aged 18–70 years with a documented history of alcohol use disorder according to DSM-5-TR [22] or an alcohol-related disorder within ICD-10 category F10, as documented in the clinical record [23], at least 12 consecutive months of continuous alcohol abstinence before enrollment/current T2 assessment, and an archived pre-enrollment MCMI-IV profile available for retrospective extraction. The 12-month criterion refers specifically to alcohol. Any alcohol consumption interrupts continuous abstinence and restarts the qualifying 12-month interval from the date of the last use. Abstinence is established by structured self-report and, where available and consented to, corroborated using clinical notes or Club-facilitator documentation. Participants must be able to understand study procedures and provide written informed consent in Romanian.

Exclusion criteria are: (1) an acute psychiatric condition requiring emergency intervention at screening, including acute psychosis or mania; (2) a current uncontrolled non-alcohol substance use disorder, defined for this study as ongoing use associated with intoxication or withdrawal, urgent treatment need, marked functional impairment, or an investigator judgment that reliable consent or study completion would be compromised; (3) legal incapacity or inability to provide written informed consent; and (4) severe cognitive impairment or communication difficulty that, in the judgment of the screening clinician, precludes valid consent or reliable completion of study procedures. Historical or stable treated use of nicotine, prescribed medication, or a non-alcohol substance disorder in sustained remission does not constitute automatic exclusion.

Recruitment uses non-probability consecutive sampling among eligible individuals attending ACAR–Don Orione services or identified through affiliated Institute of Psychiatry Socola referral pathways. The target enrollment is 70 participants, within the ethics-approved range of 60–80. During the recruitment period, approximately 210 potentially eligible beneficiaries are active across the participating ACAR setting and affiliated groups, with approximately 40 available for assessment in a typical month. These figures describe the accessible population and do not represent an assumed recruitment rate. Reasons for non-eligibility, refusal, and incomplete paired MCMI-IV data will be recorded in the study flow diagram.

### 2.4. Procedures and Timeline

Recruitment occurs at the current T2 study visit after an individual has completed at least 12 consecutive months of continuous alcohol abstinence and meets all other eligibility criteria. Potential participants receive a written information sheet and a minimum 48 h reflection period. Consent is obtained individually in a private room by trained personnel who are not responsible for the participant’s routine care. Voluntariness and the right to withdraw without consequences for ongoing care are explicitly emphasized.

From an ethics perspective, this sequencing was considered preferable because individuals at admission to residential recovery care, before achieving a stable abstinence interval, may be more vulnerable due to recent detoxification, psychological instability, acute distress, fluctuating motivation, and heightened dependency on care providers. The approved protocol therefore limits research enrollment to a later, more stable stage of recovery in order to better protect voluntariness, support autonomous decision-making, and minimize potential burden or harm associated with study participation.

**Figure 2:**
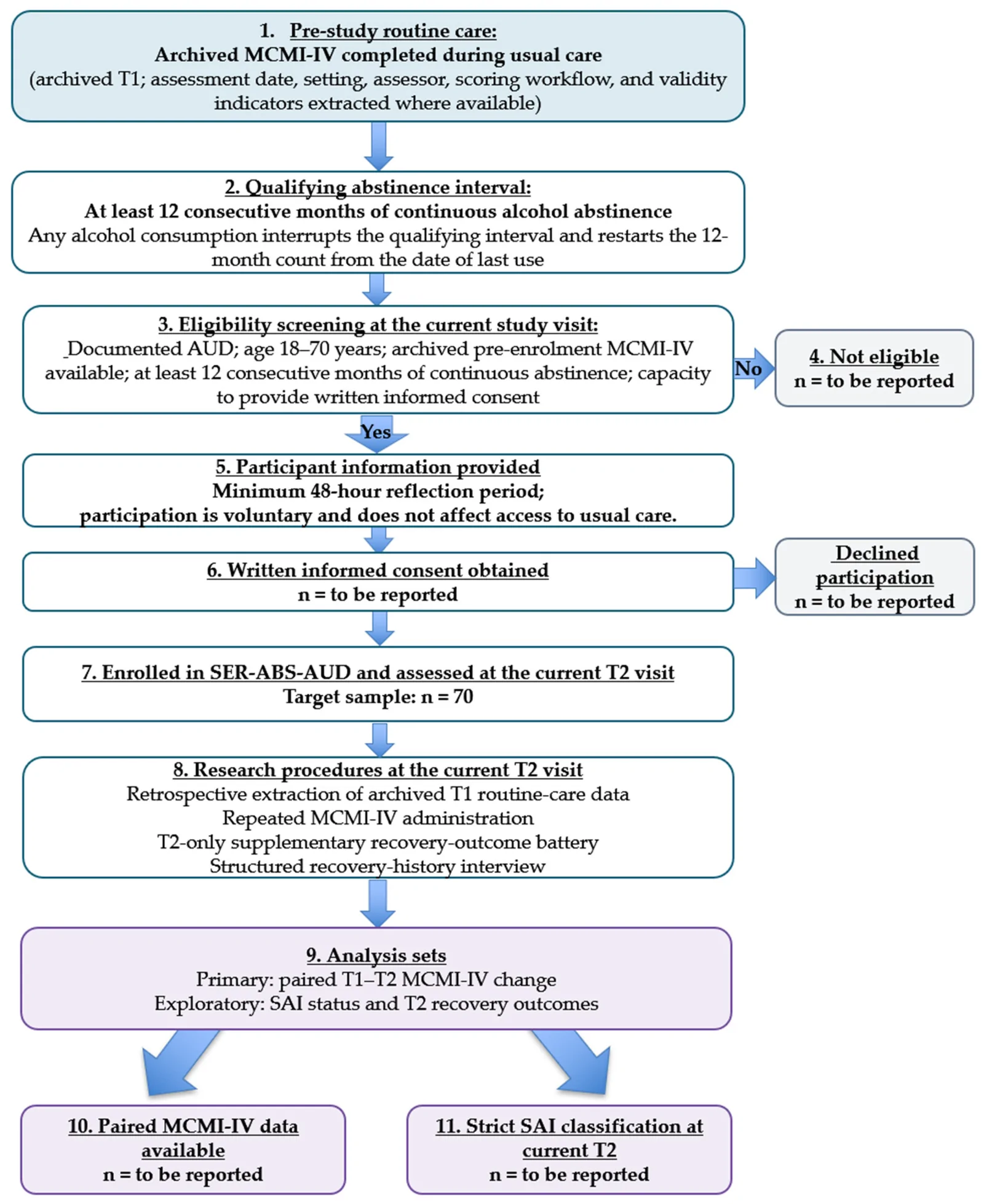
Participant flow and assessment timing in the SER-ABS-AUD cohort. Archived T1 denotes the routine-care MCMI-IV assessment conducted before study enrollment. Current T2 denotes the single research enrollment visit, performed after at least 12 consecutive months of continuous alcohol abstinence. The MCMI-IV is repeated at T2; all supplementary recovery measures are administered at T2 only. No post-enrollment 12-month follow-up visit is undertaken under the present protocol. MCMI-IV, Millon Clinical Multiaxial Inventory-IV; SAI, Serene Abstinence Index.

After consent, archived T1 data are extracted from routine-care records. The archived T1 dataset comprises the available MCMI-IV profile, assessment date, administration setting, assessor, scoring method, completion status, and validity indicators, together with eligible clinical information documented before enrollment. At the same current study visit, participants complete the T2 MCMI-IV and the prospective recovery-outcome battery over approximately 90–120 min. No standardized supplementary research measures were administered at archived T1. Retrospectively reconstructed recovery-history information is used for contextual characterization only and is not treated as psychometrically equivalent to prospectively collected T2 measures (Figure 2).

The principal longitudinal comparison is therefore the within-person difference between an archived routine-care MCMI-IV assessment and a current research MCMI-IV assessment. The identical instrument is used, but the assessment contexts may differ in clinical indication, setting, assessor, participant familiarity, elapsed abstinence duration, and scoring workflow. These features will be extracted where available, summarized, and considered in sensitivity analyses. The current T2 assessment is the enrollment assessment; participants are not recontacted 12 months after study entry under the present protocol.

The 12-month recovery journey preceding T2 is documented using a structured chronology covering engagement with recovery services, residential care, peer-support attendance, psychotherapy where applicable, major medical or psychiatric events, medication changes, employment and family transitions, and the beginning of the current uninterrupted abstinence interval. These variables are descriptive or exploratory and will be analyzed only where their definition and completeness permit. No qualitative thematic analysis is planned in this protocol. A future mixed-methods study may use separate consent, ethics approval, audio-recording procedures, and a prespecified qualitative analytic framework.

### 2.5. Measures

Measures are organized according to assessment timing and analytic role. MCMI-IV is the only standardized instrument available at both archived T1 and current T2; within-person longitudinal change is therefore evaluated only for its prespecified scales. PHQ-9, QOLI, SWLS, MOS-SSS, MMSE, the adapted spiritual-well-being item set, natural reward response, and the purpose-built biopsychosocial–spiritual questionnaire are administered prospectively at T2 only. They are analyzed as current outcomes or concurrent exploratory correlates and are not represented as T1–T2 changes.

The secondary exploratory SAI combines change in MCMI-IV persistent depression (D) with three current T2 components: natural reward response, spiritual well-being, and QOLI. Variables included in the SAI will not be entered as predictors of that same composite. All eligible archived T1 variables will be reported transparently in prespecified univariable exploratory analyses; subject to the observed outcome distribution, the restricted adjusted model will contain T1 generalized anxiety (A), T1 avoidant (2A), and duration of continuous abstinence before enrollment. Current T2 measures will be examined only as concurrent correlates of outcomes that do not contain the same variable or construct.

#### 2.5.1. Personality and Clinical Syndromes (MCMI-IV)

Personality patterns and clinical syndromes are assessed using the Romanian-language Millon Clinical Multiaxial Inventory-IV (MCMI-IV). The archived pre-enrollment MCMI-IV profile is retrieved retrospectively at T1 from routinely collected clinical records, and the same instrument is administered prospectively at T2. The prespecified scales of interest are alcohol use (Scale B), persistent depression (Scale D), generalized anxiety (Scale A), avoidant personality pattern (Scale 2A), dependent personality pattern (Scale 3), and masochistic personality pattern (Scale 8B). These scales were selected because of their relevance to alcohol-use severity, persistent depressive symptom burden, anxiety, social withdrawal, dependency-related interpersonal difficulties, and self-defeating interpersonal patterns. Standard base-rate (BR) scoring is used. Scores of ≥75 BR are interpreted as clinically notable elevations in accordance with the MCMI-IV manual; they are not used in isolation to establish a psychiatric diagnosis. Scale B will be retained primarily as a descriptive alcohol-use severity indicator. T1 persistent depression (D) will not be entered as an independent predictor of SAI because T1–T2 change in D is part of the composite. Prespecified univariable analyses will report associations of eligible archived T1 MCMI-IV scales with strict SAI status. If the event-count rule is met, the restricted adjusted model will include T1 generalized anxiety (A), T1 avoidant personality-pattern (2A), and duration of continuous abstinence before enrollment.

#### 2.5.2. Biopsychosocial–Spiritual Battery

A purpose-built, four-dimension supplementary questionnaire captures biological, psychological, social, and spiritual functioning at T2. These domain scores are exploratory current outcomes or concurrent correlates; none is treated as a validated scale or as a prospectively measured baseline predictor. Item content, response options, scoring rules, reverse-coded items, and domain-score calculations will be provided in Supplementary Table S1. Internal consistency will be reported using McDonald’s omega and, for comparability, Cronbach’s alpha where appropriate.

Biological dimension: sleep quality, energy and motivation, self-rated physical functioning, exercise frequency, cognitive clarity, and craving intensity. Items will be Likert-scaled, with negatively phrased items, including health complaints and craving intensity, reverse-coded so that higher scores consistently indicate better current functioning.

Psychological dimension: current mood stability, self-esteem, emotional processing, locus of control, resilience to triggers, shame, self-forgiveness, and trauma processing, interpreted alongside the PHQ-9 total score. Responses to PHQ-9 item 9 are managed under the safety procedure in Section 2.7.1. The inclusion of cognitive screening and planned sex-disaggregated analyses is supported by evidence that cognitive recovery during early abstinence may be heterogeneous. In a longitudinal residential-treatment cohort, female sex and lower baseline cognitive performance were associated with non-recovery of global cognitive impairment after 6 weeks of abstinence [24].

Social dimension: current family or partner relationships, friendship quantity and quality, employment status and satisfaction, support-group involvement, and community or volunteer participation. Abstinence-supportive social networks and engagement with mutual-help groups have been associated with favorable alcohol-related and recovery outcomes [14,15]. Support-group involvement is therefore included as a core social indicator; community and volunteer participation are assessed separately as exploratory indicators of social participation.

Spiritual dimension: current purpose in life, values alignment, hope, prayer/meditation, spiritual-community involvement, connection with nature, transcendence, inner peace, forgiveness, gratitude, and religious/spiritual doubt or trauma.

Each domain score will be calculated from prespecified eligible Likert items after harmonizing item directionality and reverse-coding negatively worded items where required. A domain mean will be calculated only when at least 80% of its constituent items are complete; otherwise, the domain score will be treated as missing. Higher scores will indicate better current functioning after this prespecified recoding.

This multidimensional recovery-outcome framework is consistent with longitudinal research in residential and community-based addiction-recovery settings, where recovery has been assessed not only through substance-use outcomes but also through mental health, subjective well-being, social functioning, quality of life, and employment-related indicators. In the present study, longitudinal within-person change is evaluated for MCMI-IV scales only; supplementary biopsychosocial–spiritual dimensions are interpreted as prospectively assessed T2 recovery outcomes [10].

#### 2.5.3. Supplementary Standardized and Exploratory Measures

At T2, participants complete the PHQ-9, QOLI, SWLS, MOS-SSS, MMSE, and an adapted eight-item spiritual-well-being item set. The validated status, licensing requirements, Romanian-language version, scoring method, and analytic role of each measure will be reported separately. The adapted spiritual item set is an exploratory measure and will not be presented as the validated full FACIT-Sp instrument. The adapted FACIT-Sp item set is not treated as a diagnostic instrument. Its item composition, scoring procedure, adaptation process, and internal consistency will be documented in the supplementary material and evaluated in the study sample.

These supplementary instruments are administered at T2 only and are interpreted as current recovery outcomes or concurrent correlates. They are not treated as standardized repeated T1–T2 measures. At T1, the archived pre-enrollment MCMI-IV profile is extracted from routinely collected clinical records, together with retrospectively reconstructed selected recovery information. The primary longitudinal within-cohort analysis therefore examines paired MCMI-IV change from T1 to T2; T2 QOLI, SWLS, MOS-SSS, PHQ-9, and the adapted eight-item spiritual well-being score are evaluated as current recovery indicators and concurrent exploratory correlates.

### 2.6. Outcomes

#### 2.6.1. Secondary Exploratory Outcome: Serene Abstinence Index

Participants will be classified as meeting the strict exploratory Serene Abstinence Index (SAI) definition if all four prespecified criteria are met at the current T2 assessment. Continuous abstinence during the 12 months preceding enrollment is an eligibility criterion for the study and is established by participant self-report and, where available, corroborating Club or clinical documentation. The SAI is intended to distinguish qualitatively different recovery experiences among participants meeting this abstinence criterion; it is not a diagnostic category or a validated clinical endpoint.

Persistent depression change: A reduction of at least 15 base-rate points on the MCMI-IV persistent depression (D) scale between the archived pre-enrollment assessment and T2. To indicate a clinically meaningful reduction in persistent depressive symptom burden, participants must also have an archived D score of at least 75 BR and a T2 D score of 60 BR or lower.

Natural reward response: A score of at least 4 on a 1–5 Likert item assessing enjoyment of ordinary non-alcohol-related activities at T2. Higher scores indicate greater current capacity to experience pleasure from everyday natural rewards.

Spiritual well-being: A total score of at least 26 out of 32 on the eight-item adapted spiritual well-being item set administered at T2. Each item is scored from 0 (“not at all”) to 4 (“very much”), with higher scores indicating greater current spiritual well-being. This threshold corresponds approximately to endorsement at the upper range of the response scale and is exploratory.

Quality of life: A total QOLI score of at least 3.6 at T2. QOLI is administered only at T2 and is therefore interpreted as a measure of current subjective quality of life, not as a measure of within-person quality-of-life improvement. This threshold is investigator-defined and exploratory; the continuous QOLI total score, its distribution, and the proportion of participants meeting the threshold will all be reported. QOLI will be administered and scored in accordance with the instrument manual by an appropriately trained and licensed investigator, A.-M.G., registration number MN-00020237202306-28.

The SAI is an investigator-defined, exploratory composite intended to characterize variation in recovery experience among participants who already satisfy the eligibility criterion of at least 12 consecutive months of continuous alcohol abstinence. It is not a diagnostic category, validated endpoint, or clinical prediction tool. Participants meet the strict SAI definition only when all four prespecified criteria are satisfied: (1) archived T1 MCMI-IV persistent depression (D) ≥ 75 BR, current T2 D ≤ 60 BR, and a reduction of ≥15 BR points; (2) T2 natural reward response ≥ 4 on a 1–5 Likert item; (3) T2 spiritual well-being ≥ 26/32 on the adapted eight-item set; and (4) T2 QOLI total score ≥ 3.6.

The participant-reported item contrasting feeling happy and fulfilled most days with just managing to stay sober will be used only to describe concordance with the strict classification; it will not be described as validation. Sensitivity analyses will examine fulfillment of at least three of four components, each component separately, and a continuous standardized composite if its directionality and scaling are finalized in the statistical analysis plan before database lock. The continuous measures will also be reported to avoid presenting recovery as an inherently binary phenomenon (Table 1).

#### 2.6.2. Other Secondary and Exploratory Outcomes

1. T2 biological-functioning domain score.
2. T2 psychological-functioning domain score, interpreted alongside PHQ-9.
3. T2 social-reintegration domain score.
4. T2 spiritual-domain score from the purpose-built questionnaire, reported separately from the adapted eight-item spiritual-well-being score.
5. T2 QOLI total score and SWLS total score.
6. T2 MOS Social Support Survey score.
7. T2 PHQ-9 total score.
8. T2 MMSE score.
9. Structured descriptors of the pre-enrollment recovery journey, including service engagement, major clinical or psychosocial events, and the start date of the current uninterrupted alcohol abstinence interval, where available.

All outcomes in items 1–8 are measured at current T2 only and are not interpreted as longitudinal change. Item 9 is retrospective contextual information and not a prospective outcome.

#### 2.6.3. Alcohol Lapse and Relapse

Continuous alcohol abstinence is an eligibility criterion rather than a study outcome. For eligibility, any alcohol consumption—regardless of amount or duration—interrupts the qualifying interval and restarts the 12-month count from the date of the last use. A sustained return to alcohol use may additionally be described clinically as relapse, but this distinction does not alter the eligibility rule. Alcohol-use episodes occurring before the beginning of the final qualifying 12-month interval may be recorded as recovery-history variables; they will not be analyzed as events occurring during a prospective study follow-up.

### 2.7. Data Management and Quality Assurance

Participants may experience psychosocial vulnerabilities related to AUD, including stigma, prior trauma, and, in some cases, limited socioeconomic resources. Additional safeguards are incorporated into the study procedures in accordance with the approved protocol and applicable research-ethics requirements: informed consent is obtained individually in a private setting rather than during a group recruitment session; participants retain the unconditional right to skip any question without justification; and immediate access to psychological or psychiatric support is available on request throughout data collection. In designing the final ethics-approved version of the protocol, particular attention was given to the increased vulnerability of individuals at the point of admission to residential recovery care, when recent alcohol-related instability, emotional distress, and dependency on support structures may be most pronounced. For this reason, the study does not recruit participants at entry into the center or during the earliest abstinence phase. Instead, enrollment is deferred until participants have completed at least 12 consecutive months of continuous alcohol abstinence. This approach was considered the most appropriate way to balance scientific aims with the ethical principles of respect for autonomy and non-maleficence, by reducing the risk of perceived pressure, protecting decision-making capacity in a more stable phase of recovery, and avoiding unnecessary research burden during a clinically fragile period.

Each participant receives a unique study identification code; identifying information is stored in a separate encrypted contact file accessible only to the principal investigator and specifically authorized study personnel when necessary for participant contact, safety procedures, or protocol oversight. The doctoral supervisor will access pseudonymized data only, unless access to identifiable information is specifically required by the approved protocol and institutional oversight procedures. Questionnaire data are entered into a secure, password-protected database. Study data are pseudonymized, with the re-identification key stored separately and accessible only to authorized study personnel. Pseudonymized research data will be retained for five years after publication or study completion, whichever occurs later, in accordance with the study data-management plan and applicable institutional retention requirements.

Data processing is conducted in accordance with Regulation (EU) 2016/679 (GDPR), including the principles of data minimization, integrity and confidentiality, and storage limitation [25].

A.H. has completed formal MCMI-IV training and certification (MMN-0018939/2022-12-08). Before undertaking independent study assessments, all assessors will complete protocol-specific training based on a written operations manual, standardized scripted instructions, mock assessments, and a competency checklist. The first 10 study assessments performed by each assessor will be directly supervised by the study coordinator or a delegated senior investigator. Thereafter, a randomly selected sample of at least 10% of assessment files per assessor will undergo independent quality review for consent documentation, eligibility, administration completeness, item entry, scoring output, and adherence to the protocol. Discrepancies and protocol deviations will be documented in a dated audit trail, corrected where appropriate, and addressed through retraining; recurrent discrepancies will trigger renewed supervision. These procedures constitute the prespecified cross-check of protocol fidelity across assessors. The final study report will summarize the number of files audited, the types of discrepancies or deviations identified, and the corrective actions taken. Potential participant distress during interviews will be managed through sensitive communication, the option to skip any question, and immediate referral to psychological or psychiatric support when indicated.

MCMI-IV responses are scored through the authorized standardized procedure specified in the operations manual. For any participant with documented clinician–assessor overlap, item entry, validity indicators, and resulting BR scores are independently checked by a qualified team member who is not provided with archived T1 scores, supplementary T2 outcomes, or derived SAI status. The original scoring output, verification record, role-overlap status, and any protocol deviation are retained in the study audit trail.

This study does not involve any experimental pharmacological or psychological intervention. Participants continue to receive usual ACAR Don Orione recovery care as documented above (Section 2.2); this care is not allocated, modified, or standardized by the research protocol. Ethical oversight was obtained as described in Section 2.1.

#### 2.7.1. Safety Monitoring and Research-Assessment Discontinuation

The PHQ-9 is administered during the current T2 assessment. Any response other than ‘not at all’ to PHQ-9 item 9 prompts immediate evaluation by a qualified psychiatrist or psychologist using the local suicide-risk procedure. A positive item 9 response is a screening signal and does not, by itself, establish acute suicide risk. If clinical assessment identifies acute suicide risk, severe psychiatric instability, or an immediate need for emergency care, the research assessment is stopped, and the participant is referred through the local emergency mental-health pathway. Previously collected data are retained or withdrawn in accordance with the consent form, the participant’s wishes, and applicable ethics requirements. Missing T2 data are handled under the prespecified missing-data plan. Non-acute participants may complete or resume assessment only if judged clinically appropriate. All triggers, clinical decisions, referrals, discontinuations, and adverse events related to research procedures are documented in the safety log.

#### 2.7.2. Bias Minimization

Consecutive recruitment is used to reduce discretionary selection, although the cohort may overrepresent individuals engaged in structured recovery. Consent is obtained privately after a minimum 48 h reflection period. Abstinence eligibility and recovery history are assessed through a structured interview and corroborated where available and consented to. Archived T1 data were generated in routine care, whereas T2 data are collected for research; this context difference may introduce information bias and cannot be eliminated by use of the same instrument. Available metadata on date, setting, assessor, scoring workflow, completion, and MCMI-IV validity indicators will be summarized. Sensitivity analyses will restrict paired comparisons to records meeting prespecified completeness and validity criteria and, where sample size permits, will exclude participants with clinician–assessor overlap at T2. Data-entry checks, independent audits, pseudonymization, and double entry of a random subset are used to reduce processing errors.

### 2.8. Statistical Analysis

Participant characteristics will be summarized using mean and standard deviation or median and interquartile range for continuous variables, and frequency and percentage for categorical variables. Screening, eligibility, refusal, enrollment, availability of archived T1 MCMI-IV data, and completeness of current T2 assessment will be reported. Distributional form, missingness, outliers, and potential floor or ceiling effects will be examined before inferential analyses.

#### 2.8.1. Primary Longitudinal Analysis

Within-person T1–T2 change will be evaluated only for prespecified MCMI-IV scales. T1, T2, and paired-change estimates will be reported with 95% confidence intervals and standardized paired effect sizes. Paired *t*-tests or Wilcoxon signed-rank tests will be used according to distributional properties, with emphasis on effect estimates rather than isolated *p*-values. The actual T1–T2 interval and available assessment-context variables will be described. Sensitivity analyses will apply prespecified MCMI-IV completeness and validity criteria and, if feasible, exclude documented clinician–assessor overlap.

#### 2.8.2. SAI Description and Association Analyses

The proportions meeting strict 4/4 and alternative ≥ 3/4 SAI definitions will be reported with exact 95% confidence intervals. Each SAI component and any prespecified continuous composite will also be summarized. Eligible archived T1 clinical and MCMI-IV variables will be examined individually in transparent univariable exploratory analyses, and all estimated associations—not only nominally significant findings—will be reported with 95% confidence intervals. Multiplicity and the risk of false-positive findings will be acknowledged; no variable will be selected for the adjusted model solely on the basis of its univariable *p*-value.

#### 2.8.3. Restricted Adjusted Analysis

Subject to the event-count rule specified in Section 2.8.6, Firth penalized logistic regression will estimate associations of strict SAI status with T1 generalized anxiety (A), T1 avoidant (2A), and duration of continuous abstinence before enrollment. T1 persistent depression (D) is excluded because its T1–T2 change is included in SAI. Current T2 natural reward response, spiritual well-being, and QOLI are excluded because they are SAI components. Results will be presented as exploratory odds ratios with 95% confidence intervals; no claims of model calibration, discrimination, clinical prediction performance, causal effect, or clinical utility will be made.

#### 2.8.4. Concurrent T2 Analyses

PHQ-9, QOLI, SWLS, MOS-SSS, MMSE, natural reward response, and the purpose-built domain scores will be analyzed as current T2 outcomes or concurrent correlates. A T2 variable will not be analyzed as a correlate of an outcome that contains the same variable or construct. Correlation coefficients and regression estimates will be accompanied by 95% confidence intervals and interpreted without temporal or causal language.

#### 2.8.5. Missing Data

Missingness will be reported for each source and variable. Primary paired analyses will use participants with valid paired values for the relevant scale. Characteristics available before T2 will be compared descriptively between participants with and without complete paired data. Multiple imputation by chained equations will be considered only if the amount and pattern of missingness, sample size, and available auxiliary variables support a stable and clinically plausible model under a missing-at-random assumption; otherwise, complete-case and descriptive sensitivity analyses will be reported. The likely direction of selection and missing-data bias will be discussed.

#### 2.8.6. Reporting

The results paper will follow STROBE and, for archived routine-care data, RECORD [19,20]. Analyses specified after inspection of outcome distributions will be labeled post hoc. The protocol, dated statistical analysis plan, registry history, and deviations from planned analyses will be made transparent in the results manuscript.

The Serene Abstinence Index (SAI) is a prespecified exploratory composite endpoint.

The target enrollment of 70 is pragmatic, and the strict SAI prevalence is unknown because its four components have not previously been collected jointly in this setting. For transparency, strict-SAI prevalences of 10%, 20%, 30%, and 40% in a sample of 70 would correspond to approximately 7, 14, 21, and 28 participants, respectively; these are arithmetic feasibility scenarios, not anticipated rates.

All eligible archived T1 variables will first be described and may be examined in univariable exploratory analyses with both positive and negative findings reported. The three-variable Firth penalized logistic-regression model will be fitted only if at least 20 participants meet the strict SAI definition, at least 10 participants do not meet it, and model estimation is numerically stable. With 10–19 strict-SAI events, analyses will be restricted to descriptive estimates, univariable models, or a single-predictor penalized model. With fewer than 10 strict-SAI events, strict-SAI analyses will be descriptive only.

Confidence intervals and the observed outcome counts will be emphasized throughout. Alternative SAI definitions will remain sensitivity analyses and will not be selected post hoc to obtain a more favorable event count.

## 3. Planned Analyses and Reporting

As SER-ABS-AUD is an ongoing longitudinal study, this article reports the protocol rather than outcome data. This section describes the planned reporting structure for the primary analysis.

### 3.1. Planned Descriptive Reporting

A table in the subsequent results publication will report participant flow and cohort characteristics, including the accessible recruitment pool where verifiable, screened and eligible counts, refusals, enrollment, archived-record availability, age, sex, education, occupational status, duration of continuous abstinence before enrollment, AUD-related complications, psychiatric comorbidity, service engagement, and available T1 assessment-context information.

### 3.2. Planned Longitudinal Analyses

The subsequent results publication will report archived T1 and current T2 values, paired change, 95% confidence intervals, and standardized paired effect estimates for MCMI-IV D, A, 2A, 3, and 8B; Scale B will be descriptive. Exploratory subgroup descriptions by strict SAI status will be clearly labeled and will not redefine the primary analysis.

### 3.3. Planned T2 and SAI Reporting

The subsequent results publication will report current T2 PHQ-9, QOLI, SWLS, MOS-SSS, MMSE, natural reward response, adapted spiritual-well-being score, and purpose-built domain scores as current recovery outcomes. It will also report strict and sensitivity SAI prevalence, component distributions, participant-summary-item concordance, and observed outcome counts.

### 3.4. Planned Exploratory Predictor Analyses

A separate table will report all prespecified univariable archived-T1 associations with strict SAI status, including positive and negative findings. If the event-count rule is met, the restricted Firth model will report T1 A, T1 2A, and duration of continuous abstinence. A separate section will report concurrent T2 associations only for non-overlapping outcomes. No analysis will be described as demonstrating causality, validation, or clinical prediction performance.

Use of AI-Assisted Technologies: Grammarly (Grammarly Inc., version 1.2.291) was used solely for English-language proofreading, including grammar, spelling, punctuation, and readability checks of this manuscript. It was not used for study data generation, data analysis, statistical analysis, interpretation of findings, or development of study conclusions. All scientific content, methodological decisions, analyses, and interpretations were developed and verified by the authors.

## 4. Discussion

SER-ABS-AUD addresses the need to characterize recovery from alcohol use disorder beyond alcohol-use status alone. By combining longitudinal MCMI-IV assessment with a multidimensional biopsychosocial–spiritual assessment at current T2 in a community-based recovery setting, the study will explore the feasibility and clinical interpretability of the proposed construct of “serene abstinence.” The Serene Abstinence Index is an exploratory composite endpoint, and any proposed definition will require replication, refinement, and external validation in independent Romanian and international cohorts before wider application.

This multidimensional approach is supported by evidence that apathy may occur in alcohol-related cognitive disorders, including among individuals with AUD without severe global cognitive impairment, and that behavioral and social apathy are associated with poorer everyday functioning [26]. Longitudinal evidence further indicates that cognitive recovery during abstinence is heterogeneous and may be related to baseline cognitive status and sex. Accordingly, MMSE-derived information will be retained as an exploratory clinical variable and will not be interpreted as a diagnostic assessment of alcohol-related neurocognitive disorder [24].

If exploratory analyses identify associations between archived T1 MCMI-IV profiles and strict SAI status at current T2, the findings will generate hypotheses about recovery phenotypes that may warrant prospective testing. They will not establish that MCMI-IV scores predict future recovery because T2 is the enrollment assessment, the T1 data were collected in routine care, and the composite is unvalidated. Current T2 social support, spiritual well-being, quality of life, depressive symptoms, and natural reward response will be interpreted as concurrent recovery characteristics rather than antecedent predictors.

The study may also provide descriptive information on current social support, spiritual well-being, quality of life, depressive symptoms, and natural reward response among adults engaged in community-based recovery. Because these supplementary measures are collected at T2, their associations with recovery outcomes will be interpreted as concurrent exploratory correlates rather than evidence that social or spiritual functioning prospectively predicts serene abstinence. Findings will nevertheless be relevant to the continued development of integrated recovery pathways in Romania, including long-standing community and peer-support models such as the Hudolin Club approach in Iași.

### 4.1. Neurological Comorbidity and the Biopsychosocial–Spiritual Equilibrium

A clinically relevant feature of the ACAR “Don Orione” recovery setting is its multidisciplinary orientation, incorporating psychological, psychiatric, social, and spiritual support, with referral to neurological assessment when clinically indicated. This is pertinent because alcohol use disorder may be associated with peripheral neuropathy, cognitive impairment, and withdrawal-related seizures, each of which can affect daily functioning and requires appropriate clinical assessment and follow-up [27].

Accordingly, SER-ABS-AUD documents retrospectively available and self-reported alcohol-related neurological history and includes MMSE cognitive screening at T2, alongside MCMI-IV indicators of dysthymia and anxiety and supplementary biopsychosocial–spiritual measures. The MMSE will be interpreted as an exploratory cognitive screening measure and not as a diagnostic assessment of alcohol-related neurocognitive disorder.

Quality of life is assessed only at the current T2 study visit. Accordingly, any observed association between QOLI and MCMI-IV change, social support, spiritual well-being, depressive symptoms, or other recovery characteristics will be interpreted as a concurrent exploratory association. The present design cannot determine whether these factors preceded, caused, or resulted from current subjective quality of life, and it cannot estimate improvement in quality of life over time.

Among participants with documented or self-reported alcohol-related neurological complications, exploratory analyses will describe psychological outcomes, current social and spiritual functioning, and self-reported biological or neurological health. Any observed associations will be interpreted cautiously. Because the study is observational, relies partly on retrospective and self-reported clinical information, and assesses supplementary recovery measures at T2, it cannot establish that social or spiritual functioning improves neurological recovery, treatment adherence, relapse risk, or other clinical outcomes.

### 4.2. The Socola–ACAR Collaboration: A Model of Detoxification, Aftercare, and Peer Support

The institutional pathway underlying SER-ABS-AUD links detoxification at the Institute of Psychiatry “Socola”, Iași, with post-cure residential rehabilitation and indefinite Club attendance within ACAR “Don Orione”. It represents one of the few continuously operating recovery pathways in Romania accessible irrespective of a beneficiary’s socioeconomic status. Following the 2025 restructuring of the National Antidrug Agency, which transferred addiction-related psychiatric assistance into public sanitary units and mental health and addiction prevention centers (CSMPA), ethical oversight of community-based alcohol recovery research in the Iași region has been anchored under the Ethics Committee of Institute “Socola” (approval no. 34565/17.12.2025), which explicitly recognizes ACAR as an operating partner for the present study.

The study setting links post-detoxification referral pathways with a community-based recovery environment, enabling longitudinal assessment across clinical, psychological, social, and spiritual dimensions of recovery.

This organizational feature may reduce financial barriers to participation and support the inclusion of participants from diverse socioeconomic backgrounds. However, socioeconomic characteristics and access to other health and social services will be documented where available and considered as potential confounders in the interpretation of recovery trajectories.

### 4.3. The ACAR “Don Orione” Program: Structure, Method, and the Interclub Network

The institutional pathway underlying SER-ABS-AUD links detoxification care at the Institute of Psychiatry “Socola,” Iași, with post-cure residential rehabilitation and ongoing Club participation within ACAR “Don Orione.” The study examines recovery within this real-world continuum of care, encompassing post-detoxification referral, community-based support, and longer-term peer engagement.

ACAR “Don Orione” provides residential and community-based recovery support through structured family involvement, Club participation, peer support, and spiritual-community activities. These services constitute usual care and are neither allocated nor standardized by the research protocol. Their description is provided to contextualize the service environment, not to imply that the study evaluates their effectiveness.

### 4.4. The Absence of a National Alcohol Program in Romania

As outlined in Section 1, Romania lacks a clearly identifiable, alcohol-specific national continuum-of-care program integrating prevention, treatment, and long-term recovery; alcohol-related responsibilities remain distributed across broader public-health, mental-health, addiction, fiscal, road-safety, and public-order frameworks. Existing alcohol-related measures are distributed across fiscal, road-safety, public-order, and broadcasting frameworks rather than coordinated through a dedicated continuum-of-care strategy aligned with the objectives of the WHO Global Alcohol Action Plan 2022–2030. The 2025 restructuring of the former National Antidrug Agency into the National Agency for Policies and Coordination in the Field of Drugs and Addictions (ANPCDA), including the development of mental health and addiction prevention centers (CSMPA), may create opportunities for broader addiction-service coordination. However, the practical organization, coverage, and alcohol-specific integration of these services require continued assessment.

Within this context, the Socola–ACAR pathway illustrates a locally established hospital-to-community recovery arrangement linking detoxification, post-cure rehabilitation, and continuing peer-support attendance. SER-ABS-AUD does not evaluate the effectiveness of this pathway or of the ACAR/Hudolin model. Rather, it will characterize MCMI-IV trajectories and biopsychosocial–spiritual recovery characteristics among people already engaged in this service context. The findings may generate hypotheses relevant to future alcohol-service planning, particularly regarding continuity between detoxification, psychiatric follow-up, social support, and sustained community recovery resources.

### 4.5. Limitations

This study has several anticipated limitations. First, consecutive non-probability recruitment from a single recovery network may overrepresent individuals who remain engaged in structured support and who have already achieved at least 12 months of continuous abstinence. Selection and survivorship bias will therefore limit generalizability to people with active AUD, shorter abstinence, or limited access to recovery services. This ethics-driven design choice may further limit generalizability to individuals in the earliest and most clinically fragile phases of recovery, but it was considered preferable in order to avoid recruiting participants at a stage of heightened vulnerability.

Second, archived T1 MCMI-IV data were collected in routine care, whereas T2 MCMI-IV data are collected prospectively for research. Even with the same instrument, differences in clinical indication, setting, assessor, participant familiarity, elapsed abstinence duration, and scoring workflow may contribute to observed change. Assessment-context metadata and validity indicators will be reported where available, and sensitivity analyses will be performed, but these procedures cannot establish measurement equivalence or remove information bias. RECORD-guided reporting will improve transparency but cannot correct limitations inherent in secondary use of routine-care data.

Third, retrospective reconstruction of the recovery journey and self-reported abstinence may be affected by incomplete documentation, recall, and social-desirability bias. Corroboration with clinical or Club records will be used where available and consented to, but not all events will have an independent source. Because continuous alcohol abstinence is an eligibility criterion, the study cannot estimate relapse incidence during the qualifying interval or among people who did not remain abstinent long enough to enroll.

Fourth, the strict SAI may be uncommon in a sample of approximately 70. The resulting estimates may be imprecise and may preclude adjusted regression despite penalization. The SAI is investigator-defined, its thresholds are exploratory, and concordance with one participant-summary item does not constitute validation. Strict, alternative, component-level, and continuous results will therefore be reported transparently without selecting the definition that produces the most favorable result.

Fifth, most supplementary measures are collected only at current T2. Their associations are cross-sectional and cannot establish temporal direction or causality. The purpose-built domain scores and adapted spiritual-well-being item set require psychometric evaluation. Clinician–assessor overlap may remain for a subset of participants despite logging, independent score verification, and sensitivity analysis.

Finally, the study does not compare ACAR with hospital treatment or another recovery program and cannot estimate the effectiveness of ACAR, the Hudolin model, spirituality, peer support, or any other component of usual care. Future studies should use prospectively harmonized baseline and follow-up measures, independent assessors, multicentre recruitment, qualitative methods under a separate protocol, longer post-enrollment follow-up for relapse, and external validation of any SAI operationalization.

Therefore, this single-center observational design further precludes causal inference regarding the ACAR/Hudolin model, recovery setting, peer support, spirituality, or any other component of care. The study is not designed to determine whether community participation improves abstinence, neurological recovery, quality of life, or other outcomes. Findings will be interpreted within the local Socola–ACAR service environment and should not be generalized uncritically to other Romanian settings or international recovery populations.

Future research should replicate the protocol across additional Romanian recovery centers and, where feasible, in international Hudolin Club networks. Priorities include prospective measurement of social and spiritual variables from baseline, incorporation of more comprehensive neurocognitive and biological measures, longer follow-up for relapse and functional outcomes, and formal external validation of the Serene Abstinence Index.

## 5. Conclusions

SER-ABS-AUD is a single-center mixed retrospective–prospective observational cohort study combining archived routine-care MCMI-IV data with a current prospectively administered T2 assessment. Its primary analysis will estimate within-person change in prespecified MCMI-IV scales, while the SAI will be examined as a secondary, investigator-defined exploratory composite among adults who have already achieved at least 12 months of continuous alcohol abstinence. Supplementary biopsychosocial–spiritual measures collected at T2 will describe current recovery and concurrent associations only. The study is not designed to establish the effectiveness of ACAR or the Hudolin model, infer causal effects, validate a diagnostic recovery category, or develop a clinically deployable prediction model. Its findings will provide preliminary estimates and hypotheses for prospectively harmonized, independently assessed, multicentre validation studies.

## Figures and Tables

**Table 1:** Prespecified exploratory components of the Serene Abstinence Index at the current T2.

Domain	Criterion	Threshold
Psychological	Reduction in persistent depressive symptom burden MCMI-IV D from archived T1 to current T2	T1 ≥ 75 BR, T2 ≤ 60 BR, and reduction ≥ 15 BR points
Natural reward response	Single-item indicator of enjoyment of ordinary non-alcohol-related activities	≥4 on 1–5 scale
Spiritual	Adapted eight-item spiritual well-being score at T2 (exploratory; not the full validated FACIT-Sp instrument)	≥26 of 32
Quality of life	QOLI total score at T2	≥3.6
Classification rule	Strict classification rule	All 4 criteria met
Sensitivity analysis	Alternative operationalization	≥3/4 criteria met

BR, base-rate score; QOLI, Quality of Life Inventory. The adapted eight-item spiritual well-being score is an investigator-adapted exploratory item set and is not the complete validated FACIT-Sp instrument. The single natural-reward-response item is distinct from the six-item biological-functioning composite. The QOLI component is a current T2 score and not a T1–T2 quality-of-life change measure. All SAI thresholds are exploratory and unvalidated.

## Data Availability

Individual-level data generated during this study will not be publicly available because they include sensitive special-category personal data, including psychiatric history and recovery-related psychosocial information, collected from a vulnerable population. The participant-information and consent procedures and the study’s ethical and data-governance arrangements do not permit third-party sharing of individual-level data. De-identified aggregate results and summary statistics will be reported in future peer-reviewed publications. Requests for clarification regarding the study data or additional aggregate analyses may be directed to the corresponding author.

## Supplementary Materials

The following supporting information can be downloaded at: https://www.mdpi.com/article/10.3390/jcm15218260/s1, Table S1. STROBE and RECORD reporting checklists for the SER-ABS-AUD study protocol.

## Abbreviations

The following abbreviations are used in this manuscript:

AUD	Alcohol Use Disorder
ACAR	Association of Alcoholics in Recovery Clubs (Asociația Cluburilor Alcoolicilor în Recuperare)
MCMI-IV	Millon Clinical Multiaxial Inventory, 4th Edition
PHQ-9	Patient Health Questionnaire-9
FACIT-Sp	Functional Assessment of Chronic Illness Therapy—Spiritual Well-Being
SWLS	Satisfaction With Life Scale
MOS-SSS	Medical Outcomes Study Social Support Survey
QOLI	Quality of Life Inventory
BR	Base-Rate (MCMI-IV scoring metric)
GDPR	General Data Protection Regulation
STROBE	Strengthening the Reporting of Observational Studies in Epidemiology
IRB	Institutional Review Board
MMSE	Mini-Mental State Examination
SAI	Serene Abstinence Index
RECORD	REporting of studies Conducted using Observational Routinely-collected health Data

## References

[1] World Health Organization (2024). Global Status Report on Alcohol and Health and Treatment of Substance Use Disorders.

[2] Organisation for Economic Co-operation and Development (2025). OECD Reviews of Health Systems: Romania 2025.

[3] Huang J., Ahmed I.M., Wang T., Xie C. (2024). Beyond the Liver: Neurologic Manifestations of Alcohol Use. Clin. Liver Dis..

[4] Noble J.M., Weimer L.H. (2014). Neurologic Complications of Alcoholism. Continuum.

[5] Jesse S., Bråthen G., Ferrara M., Keindl M., Ben-Menachem E., Tanasescu R., Brodtkorb E., Hillbom M., Leone M.A., Ludolph A.C. (2017). Alcohol Withdrawal Syndrome: Mechanisms, Manifestations, and Management. Acta Neurol. Scand..

[6] Martinotti G., Hatzigiakoumis D.S., De Vita O., Clerici M., Petruccelli F., Di Giannantonio M., Janiri L. (2012). Anhedonia and Reward System: Psychobiology, Evaluation, and Clinical Features. Int. J. Clin. Med..

[7] Garfield J.B.B., Lubman D.I., Yücel M. (2014). Anhedonia in Substance Use Disorders: A Systematic Review of Its Nature, Course and Clinical Correlates. Aust. N. Z. J. Psychiatry.

[8] Manning V., Garfield J.B.B., Lam T., Allsop S., Berends L., Best D., Buykx P., Room R., Lubman D.I. (2019). Improved Quality of Life Following Addiction Treatment Is Associated with Reductions in Substance Use. J. Clin. Med..

[9] Farré A., Tirado J., Spataro N., Alías-Ferri M., Torrens M., Fonseca F. (2020). Alcohol-Induced Depression: Clinical, Biological and Genetic Features. J. Clin. Med..

[10] Staiger P.K., Liknaitzky P., Lake A.J., Gruenert S. (2020). Longitudinal Substance Use and Biopsychosocial Outcomes Following Therapeutic Community Treatment for Substance Dependence. J. Clin. Med..

[11] Galanter M., White W.L., Khalsa J., Hansen H. (2024). A Scoping Review of Spirituality in Relation to Substance Use Disorders: Psychological, Biological, and Cultural Issues. J. Addict. Dis..

[12] Zemore S.E. (2007). A Role for Spiritual Change in the Benefits of 12-Step Involvement. Alcohol. Clin. Exp. Res..

[13] McInerney K., Cross A. (2021). A Phenomenological Study: Exploring the Meaning of Spirituality in Long-Term Recovery in Alcoholics Anonymous. Alcohol. Treat. Q..

[14] Groh D.R., Jason L.A., Keys C.B. (2008). Social Network Variables in Alcoholics Anonymous: A Literature Review. Clin. Psychol. Rev..

[15] Kelly J.F., Humphreys K., Ferri M. (2020). Alcoholics Anonymous and Other 12-Step Programs for Alcohol Use Disorder. Cochrane Database Syst. Rev..

[16] Millon T., Grossman S., Millon C. (2015). MCMI-IV: Millon Clinical Multiaxial Inventory-IV Manual.

[17] De la Rosa-Cáceres A., Rodríguez-Testal J.F., Perona-Garcelán S., Ruíz-Veguilla M., Pérez-Gálvez B., Barragán-Ramos D., García-Montes J.M. (2022). Bridge Nodes between Personality Traits and Alcohol-Use Disorder Criteria: The Relevance of Externalizing Traits of Risk Taking, Callousness, and Irresponsibility. J. Clin. Med..

[18] Bahji A. (2024). Navigating the Complex Intersection of Substance Use and Psychiatric Disorders: A Comprehensive Review. J. Clin. Med..

[19] von Elm E., Altman D.G., Egger M., Pocock S.J., Gøtzsche P.C., Vandenbroucke J.P. (2007). The Strengthening the Reporting of Observational Studies in Epidemiology (STROBE) Statement: Guidelines for Reporting Observational Studies. Lancet.

[20] Benchimol E.I., Smeeth L., Guttmann A., Harron K., Moher D., Petersen I., Sørensen H.T., von Elm E., Langan S.M. (2015). The REporting of studies Conducted using Observational Routinely-collected health Data (RECORD) Statement. PLoS Med..

[21] ClinicalTrials.gov SER-ABS-AUD: Bio-Psycho-Socio-Spiritual Predictors of AUD Recovery. Identifier NCT07561749. NCT07561749.

[22] American Psychiatric Association (2022). Diagnostic and Statistical Manual of Mental Disorders.

[23] World Health Organization (2016). International Statistical Classification of Diseases and Related Health Problems, 10th Revision, Version 2016.

[24] Luquiens A., Rolland B., Pelletier S., Alarcon R., Donnadieu-Rigole H., Benyamina A., Nalpas B., Perney P. (2019). Role of Patient Sex in Early Recovery from Alcohol-Related Cognitive Impairment: Women Penalized. J. Clin. Med..

[25] European Parliament and Council of the European Union (2016). Regulation (EU) 2016/679 of the European Parliament and of the Council of 27 April 2016 on the Protection of Natural Persons with Regard to the Processing of Personal Data and on the Free Movement of Such Data. Off. J. Eur. Union.

[26] van Dorst M.E.G., Rensen Y.C.M., Husain M., Kessels R.P.C. (2021). Behavioral, Emotional and Social Apathy in Alcohol-Related Cognitive Disorders. J. Clin. Med..

[27] Nutt D., Hayes A., Fonville L., Zafar R., Palmer E.O.C., Paterson L., Lingford-Hughes A. (2021). Alcohol and the brain. Nutrients.

