# Supplementary material for: Neuropsychosocial–Spiritual Correlates of Serene Abstinence in Adults with Alcohol Use Disorder Receiving Community-Based Recovery Care in Romania: A Longitudinal Observational Study Protocol (SER-ABS-AUD)
Source: J Clin Med. 2026 Oct 23;15(21):8260. doi: 10.3390/jcm15218260 (PMC13763576; doi:10.3390/jcm15218260)
Supplement: Supplementary file 1 [file jcm-15-08260-s001.zip › jcm-4521105-supplementary.pdf]

Table S1. STROBE and RECORD reporting checklists for the SER-ABS-AUD study protocol

Because this is a protocol article, STROBE result-reporting items are identified as planned reporting rather than completed participant-level reporting. RECORD applies only to the archived routine-care T1 component.

Part A. STROBE Statement—checklist for cohort studies

Section/topic	Item	STROBE recommendation	Exact location in current manuscript
**TITLE AND ABSTRACT**			
Title and abstract	1(a)	Indicate the study design with a commonly used term in the title or abstract.	p. 1: title and Abstract—Methods.
	1(b)	Provide in the abstract an informative and balanced summary of what was done and what was found.	p. 1: complete structured Abstract; Results states that recruitment is ongoing and no participant-level outcome data are reported.
**INTRODUCTION**			
Background/rationale	2	Explain the scientific background and rationale for the investigation.	pp. 2–3: Section 1, Introduction, from AUD burden through the rationale for multidimensional recovery assessment.
Objectives	3	State specific objectives, including any prespecified hypotheses.	pp. 3–4: final two paragraphs of Section 1 (five objectives and principal exploratory hypothesis).
**METHODS**			
Study design	4	Present key elements of study design early in the paper.	p. 4: Section 2.1, first two paragraphs.
Setting	5	Describe the setting, locations, and relevant dates, including periods of recruitment, exposure, follow-up, and data collection.	pp. 4–6: Sections 2.1–2.2; pp. 7–8: Section 2.4 and Figure 2. Recruitment start is on p. 4; absence of post-enrolment follow-up is on pp. 7–8.
Participants	6(a)	Give eligibility criteria and sources and methods of participant selection. Describe methods of follow-up.	pp. 6–7: Section 2.3; pp. 7–8: Section 2.4. No post-enrolment follow-up is undertaken.
	6(b)	For matched studies, give matching criteria and numbers of exposed and unexposed participants.	Not applicable—the study is not matched and has no exposed/unexposed comparison groups.

Variables	7	Clearly define outcomes, exposures, predictors, potential confounders, and effect modifiers. Give diagnostic criteria, if applicable.	p. 6: Section 2.3; pp. 8–12: Sections 2.5–2.6 and Table 1; pp. 14–16: Sections 2.8 and 3.4.
Data sources/measurement	8*	For each variable, give data sources and assessment methods. Describe comparability where applicable.	pp. 7–10: Sections 2.4–2.5; pp. 12–14: Section 2.7. T1–T2 assessment-context comparability is addressed on p. 7 and in Section 2.7.2 on p. 14.
Bias	9	Describe efforts to address potential sources of bias.	pp. 5–6: Section 2.2; pp. 7–8: Section 2.4; pp. 12–14: Sections 2.7–2.7.2; pp. 18–20: Section 4.5.
Study size	10	Explain how study size was arrived at.	pp. 6–7: Section 2.3 (target n=70; ethics-approved range 60–80); p. 15: Section 2.8.6 (pragmatic target and event-count scenarios).
Quantitative variables	11	Explain how quantitative variables were handled, including groupings and rationale.	pp. 8–11: Sections 2.5–2.6.1 and Table 1; pp. 14–15: Sections 2.8.1–2.8.6.
Statistical methods	12(a)	Describe all statistical methods, including those used to control for confounding.	pp. 14–15: Sections 2.8.1–2.8.6.
	12(b)	Describe methods used to examine subgroups and interactions.	p. 9: Section 2.5.2 (planned sex-disaggregated analyses); p. 16: Sections 3.2 and 3.4 (exploratory subgroup reporting). No formal interaction analysis is prespecified.
	12(c)	Explain how missing data were addressed.	p. 15: Section 2.8.5.
	12(d)	If applicable, explain how loss to follow-up was addressed.	Not applicable—no post-enrolment follow-up. See p. 4, Section 2.1; pp. 7–8, Section 2.4 and Figure 2.
	12(e)	Describe sensitivity analyses.	p. 11: Section 2.6.1; p. 14: Sections 2.7.2 and 2.8.1; p. 15: Sections 2.8.2 and 2.8.6.
**RESULTS**			
Participants	13(a)	Report numbers of individuals at each stage.	Protocol—planned reporting: pp. 6–7, Section 2.3; p. 8, Figure 2; pp. 15–16, Section 3.1.
	13(b)	Give reasons for non-participation at each stage.	Protocol—planned reporting: p. 7, final paragraph of Section 2.3; pp. 15–16, Section 3.1.

Table S1. STROBE and RECORD reporting checklists for the SER-ABS-AUD study protocol

	13(c)	Consider use of a flow diagram.	p. 8: Figure 2; planned participant-flow reporting on pp. 15–16, Section 3.1.
Descriptive data	14(a)	Give participant characteristics and information on exposures and potential confounders.	Protocol—planned reporting: pp. 15–16, Section 3.1.
	14(b)	Indicate number of participants with missing data for each variable.	Protocol—planned reporting: pp. 14–15, opening paragraph of Section 2.8 and Section 2.8.5; pp. 15–16, Section 3.1.
	14(c)	Summarize follow-up time.	Archived-T1-to-current-T2 interval: p. 4, Section 2.1. No post-enrolment follow-up: pp. 7–8, Section 2.4 and Figure 2.
Outcome data	15*	Report numbers of outcome events or summary measures over time.	Protocol—planned reporting: pp. 14–15, Sections 2.8.1–2.8.4; p. 16, Sections 3.2–3.3.
Main results	16(a)	Give unadjusted and adjusted estimates and precision; identify adjusted variables and rationale.	Protocol—planned reporting: pp. 14–15, Sections 2.8.1–2.8.3 and 2.8.6; p. 16, Sections 3.2–3.4.
	16(b)	Report category boundaries when continuous variables were categorized.	pp. 9–11: Sections 2.5.1 and 2.6.1; pp. 11–12: Table 1 and footnote.
	16(c)	If relevant, translate relative risks into absolute risk for a meaningful time period.	Not applicable—no relative-risk model or prospective event follow-up is planned.
Other analyses	17	Report other analyses, including subgroup and sensitivity analyses.	Protocol—planned reporting: pp. 14–15, Sections 2.8.1–2.8.6; p. 16, Sections 3.2–3.4.
**DISCUSSION**			
Key results	18	Summarize key results with reference to objectives.	Not applicable at protocol stage; no participant-level results are reported. Planned interpretive framework: pp. 16–20, Sections 4–5.
Limitations	19	Discuss limitations, including potential bias or imprecision and their likely direction or magnitude.	pp. 18–20: Section 4.5.
Interpretation	20	Give a cautious overall interpretation considering objectives, limitations, multiplicity, similar studies, and other evidence.	pp. 16–20: Sections 4–5, with explicit limits on causal and predictive claims.

Generalisability	21	Discuss generalisability/external validity.	pp. 19–20: Section 4.5 and Section 5.
**OTHER INFORMATION**			
Funding	22	Give source of funding and funder role.	p. 21: Funding (“This research received no external funding.”).

Part B. RECORD extension—archived routine-care T1 component

RECORD section	Item	RECORD extension	Exact location in current manuscript
Title and abstract	1.1	Specify the type of routinely collected data in the title or abstract and, when possible, the database name.	p. 1: Abstract—Methods identifies archived pre-enrolment MCMI-IV data and available clinical information. No named external database is used.
Title and abstract	1.2	Report the geographic region and timeframe within which the study took place, if applicable.	p. 1: title and Abstract identify Romania/community-based recovery; p. 4, Section 2.1 gives the recruitment start date.
Title and abstract	1.3	Clearly state any linkage between databases in the title or abstract.	Not applicable—no linkage between separate databases is performed.
Participants	6.1	Describe methods used to select the study population, including codes or algorithms.	pp. 6–7: Section 2.3; pp. 7–8: Section 2.4. Selection uses documented DSM-5-TR/ICD-10 AUD history, abstinence eligibility, archived MCMI-IV availability, and consecutive recruitment; no database-selection algorithm is used.
Participants	6.2	Reference validation studies of codes or algorithms used to select the population; report study-specific validation.	Not applicable—no administrative-code or database algorithm is used for selection.
Participants	6.3	If databases were linked, consider a flow diagram demonstrating linkage and counts.	Not applicable—no database linkage. Assessment timing is shown in Figure 2 on p. 8.
Variables	7.1	Provide a complete list of codes and algorithms used to classify exposures, outcomes, confounders, and effect modifiers.	pp. 8–12: Sections 2.5–2.6 and Table 1 define MCMI-IV scales, SAI criteria, and supplementary outcome roles. No administrative classification algorithm is used.

Table S1. STROBE and RECORD reporting checklists for the SER-ABS-AUD study protocol

Data access and cleaning	12.1	Describe investigators' access to the database population used to create the study population.	pp. 7–8: Section 2.4 (extraction after consent); pp. 12–13: Section 2.7 (access restricted to the principal investigator and specifically authorized personnel).
Data access and cleaning	12.2	Provide information on data-cleaning methods.	pp. 12–14: Sections 2.7 and 2.7.2 (pseudonymization, standardized entry, independent review, audit trail, score verification, completeness/validity criteria, and double entry of a random subset).
Linkage	12.3	State whether linkage across two or more databases was conducted and report methods and linkage quality.	Not applicable—no linkage across separate databases. Archived T1 and current T2 data are assembled within each consented participant's study record using a unique study ID (pp. 12–13, Section 2.7).
Results—participants	13.1	Describe selection of included persons, including filtering based on data quality, availability, and linkage; use text/flow diagram.	Protocol—selection and planned reporting: pp. 6–8, Sections 2.3–2.4 and Figure 2; pp. 15–16, Section 3.1. No linkage filtering applies.
Discussion—limitations	19.1	Discuss implications of using data not created for the research question, including misclassification, unmeasured confounding, missing data, and changing eligibility over time.	pp. 19–20: Section 4.5, including routine-care/research context differences, incomplete documentation, recall/social-desirability bias, missing psychometric information, selection, and imprecision.
Access to protocol, data, and code	22.1	Provide information on access to supplementary information, protocol, raw data, or programming code.	p. 4: Section 2.1 (registry/amendments); p. 15: Section 2.8.6 (protocol, dated SAP, registry history, deviations); p. 21: Data Availability Statement (individual-level data restricted; aggregate results/analyses may be requested).

Scope note. RECORD applies specifically to the archived routine-care T1 component. Database-linkage and administrative-algorithm items are not applicable because the protocol does not link separate databases and does not use code-based population-selection algorithms.

Checklist sources: von Elm E, et al. Lancet. 2007;370:1453–1457. doi:10.1016/S0140-6736(07)61602-X. Benchimol EI, et al. PLoS Med. 2015;12:e1001885. doi:10.1371/journal.pmed.1001885.

Obtained and adapted from the EQUATOR Network internet platform (https://www.equator-network.org/)
